# Rheumatoid Arthritis and COVID-19 at the Intersection of Immunology and Infectious Diseases: A Related PRISMA Systematic Literature Review

**DOI:** 10.3390/ijms252011149

**Published:** 2024-10-17

**Authors:** Andreea-Iulia Vlădulescu-Trandafir, Violeta-Claudia Bojincă, Constantin Munteanu, Aurelian Anghelescu, Cristina Popescu, Simona-Isabelle Stoica, Sorina Aurelian, Andra Bălănescu, Cristina Băetu, Vlad Ciobanu, Gelu Onose

**Affiliations:** 1 Faculty of Medicine, University of Medicine and Pharmacy “Carol Davila”, 020022 Bucharest, Romania; andreea-iulia.trandafir@drd.umfcd.ro (A.-I.V.-T.); violeta.bojinca@umfcd.ro (V.-C.B.); sorina.aurelian@umfcd.ro (S.A.); andra.balanescu@umfcd.ro (A.B.); cristina.bcris@yahoo.com (C.B.); gelu.onose@umfcd.ro (G.O.); 2 Neuromuscular Rehabilitation Clinic Division, Teaching Emergency Hospital “Bagdasar-Arseni”, 041915 Bucharest, Romania; aurelian.anghelescu@umfcd.ro (A.A.); stoica.simona@umfcd.ro (S.-I.S.); 3 Internal Medicine and Rheumatology Departments, “Sfânta Maria” Hospital, 011172 Bucharest, Romania; 4 Faculty of Medical Bioengineering, University of Medicine and Pharmacy “Grigore T. Popa” Iasi, 700454 Iasi, Romania; 5 Faculty of Midwifery and Nursing, University of Medicine and Pharmacy “Carol Davila”, 020022 Bucharest, Romania; 6 Gerontology and Geriatrics Clinic Division, St. Luca Hospital for Chronic Illnesses, 041915 Bucharest, Romania; 7 Neurology Department, Colentina Clinical Hospital, 020125 Bucharest, Romania; 8 Computer Science Department, Politehnica University of Bucharest, 060042 Bucharest, Romania; vlad.ciobanu@upb.ro

**Keywords:** rheumatoid arthritis, COVID-19, SARS-CoV-2, PRISMA systematic literature review, Rituximab, COVID-19 vaccination

## Abstract

Rheumatoid arthritis (RA) patients face different health challenges when infected with severe acute respiratory syndrome coronavirus 2 (SARS-CoV-2) than the general population, due to both their immunocompromised state and the immunosuppressive therapies they receive. This systematic literature review, which follows the Preferred Reporting Items for Systematic Reviews and Meta-Analyses (PRISMA) paradigm, explores the interactions between RA and SARS-CoV-2 infection, focusing on immunologic issues, disease management, vaccination, and adverse outcomes. In order to obtain the most relevant information, we systematically reviewed the specific literature from 1 January 2021 to 31 December 2023, based on the PRISMA method, by which we eventually selected 35 eligible articles, to which we added other ISI-indexed studies to enrich our results further. Consequently, we performed a funnel analysis to evaluate the potential for publication bias. Firstly, the data collected revealed the impact of the pandemic on RA diagnoses and the fear of face-to-face medical consultations that delayed adequate treatment. Secondly, cardiovascular and metabolic comorbidities increase the risk of prolonged COVID-19 symptoms, hospitalization, and severe COVID-19 outcomes for RA patients. With respect to immunosuppressive treatment used to control RA, it was observed that glucocorticoids (especially high-dose usage) and Rituximab (RTX) predispose the patients to poor SARS-CoV-2 outcomes, as opposed to Baricitinib and interleukin-6 (IL-6) and tumor necrosis factor-alpha (TNF-α) inhibitors. COVID-19 vaccination has proven effective and generally safe for RA patients in some studies, although therapies with Methotrexate (MTX), Abatacept (ABA), and RTX have been associated with impaired vaccine immune response. This systematic literature review brings updated and thorough information with respect to the immunological, clinical, and management of a complex immune-mediated inflammatory disease (IMID) like RA in the setting of COVID-19 and underlines the challenges faced by this group of patients. The lessons learned can be extended beyond the pandemic in shaping a more informed and compassionate healthcare system and offering long-term medical care for patients with RA.

## 1. Introduction

The recent global health crisis known as “Coronavirus Disease 2019” (COVID-19), brought on by a strain of coronavirus—severe acute respiratory syndrome coronavirus 2 (SARS-CoV-2)—has created enormous burdens for patients, healthcare providers, whole national economies, and researchers around the world. The first case of COVID-19 was reported in Wuhan, China, in December 2019. Later, in March 2020, as the number of cases increased dramatically, the World Health Organization (WHO) officially declared this infection a global pandemic [1,2,3].

SARS-CoV-2-induced infections were a source of a lot of uncertainties for RA patients. Due to the weakened immune response characteristic of this particular group of individuals, along with their continued reliance on immunosuppressive treatments, this population is at an elevated risk for severe consequences associated with COVID-19, as indicated by various studies [4,5,6].

The pandemic led to a substantial decline in the diagnoses of RA, mainly due to patients’ reluctance to seek face-to-face medical consultations for fear of virus transmission. Many RA exacerbations were handled by the patients themselves, outside the realm of professional observation, which led to documented interruptions in disease-modifying antirheumatic drug (DMARD) administration, most of which were not recommended by health professionals [1,7,8].

With respect to COVID-19 clinical manifestations, RA patients exhibit a myriad of symptoms: constitutional/systemic, musculoskeletal, respiratory, and neurological. A significant number of these individuals may require prolonged hospitalization, with specific studies indicating that the mortality rate within 30 days can reach up to 8.6% among this population [4,9].

Another matter that is receiving significant attention pertains to the persistent symptoms of COVID-19, which may average a duration of one month and extend beyond six months. These symptoms include fatigue, dyspnea, alteration of smell (hyposmia/anosmia) and taste (ageusia/hypogeusia), and nasal congestion. Thus, COVID-19 is not merely a typical infectious disease but a serious condition impacting various organs [2,10,11].

Several factors influence the outcome of COVID-19 in RA patients, such as comorbidities, RA duration, and the type of DMARDs administered. Some of the comorbidities, such as diabetes mellitus (DM) and cardiovascular diseases (CVDs), may increase the risk for serious SARS-CoV-2 outcomes, especially with respect to length of hospitalization stay and death [11,12,13,14].

Immunosuppressive agents are of utmost importance in managing RA. However, during the COVID-19 pandemic, unforeseen results were observed with their use. For instance, GCs are highly established for their potent anti-inflammatory effects, but they might facilitate severe SARS-CoV-2 outcomes due to the high likelihood of opportunistic infections [10,15], by the global immune depression they act on (including therapeutically). Similarly, biological agents (bDMARDs), such as RTX, could render RA patients vulnerable to serious COVID-19 evolution [16,17,18]. In contrast, other bDMARDs like TNF-α and IL-6 inhibitors may reduce the severity of SARS-CoV-2 as they may prevent the cytokine storm that is believed to cause life-threatening consequences [2,9,19].

Furthermore, although immunization has been a critical strategy in the fight against COVID-19, patients with RA have unique challenges, which include impaired vaccine responses and safety concerns. Nonetheless, COVID-19 vaccines have been generally safe for RA patients, offering protection against severe SARS-CoV-2 infection. However, further studies are still required to assess vaccination effectiveness in this population [20,21,22,23,24].

The following article will outline these dynamics, emphasizing the continued need for vigilance and RA-tailored care in the setting of ongoing SARS-CoV-2 infections.

## 2. Materials and Methods

### 2.1. Study Design

**Figure 1:**
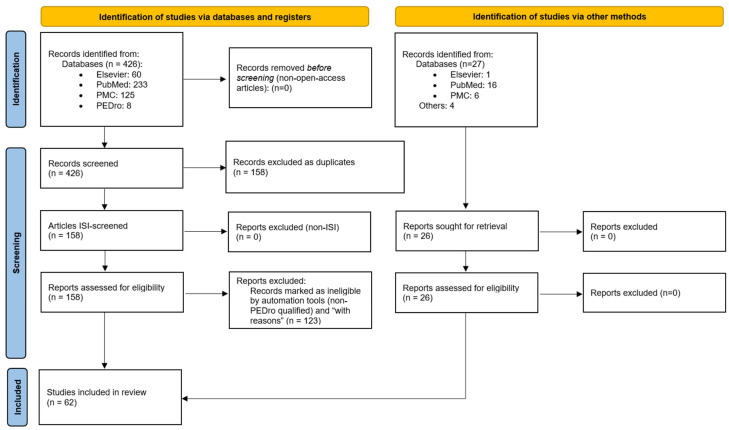
Adapted PRISMA flow diagram (2020), customized for our study.

We conducted a comprehensive systematic literature review following the PRISMA paradigm to gain deeper insights into the association between RA and COVID-19. As depicted in Figure 1, the initial phase involved a meticulous and comprehensive search for relevant articles from 1 January 2021 to 31 December 2023. This process included accessing reputable international medical databases such as the National Center for Biotechnology Information (NCBI)/PubMed (available at https://pubmed.ncbi.nlm.nih.gov/, last accessed on 19 April 2024), NCBI/PubMed Central (PMC—available at https://www.ncbi.nlm.nih.gov/pmc/, last accessed on 19 April 2024), Elsevier (available at https://www.elsevier.com/, last accessed on 19 April 2024), and Physiotherapy Evidence Database (PEDro) [25] and verifying the publication of the retrieved works in ISI-indexed journals through the Web of Science database [26,27]. In searching, we used contextually specific keyword combinations/”syntaxes”: “COVID-19”, “SARS-CoV-2”, “Novel Coronavirus Disease”, “rheumatoid arthritis”, “RA”, “symptoms”, “corticosteroids”, “DMARD”, “disease-modifying rheumatic drug”, “C Reactive Protein”, “chest imaging”, “sequelae”, “hospitalization”, “flare”, “admission risk”, “age”, “vaccination” (Appendix A).

In our initial screening, we exclusively considered articles available in open access, written in English, and hence initially identified 426 articles. Subsequently, after the removal of duplicates/redundancies, 158 articles remained on our list. These 158 papers were then scrutinized for inclusion in ISI Thomson Reuters-indexed publications, and all met this criterion. In the next stage, we utilized a custom quantification-weighted algorithm, a unique and innovative approach inspired by PEDro classification/scoring, to indirectly assess the scientific impact and quality of each of the remaining articles. Only articles achieving a minimum score of 4 (“fair quality = PEDro score 4–5”) were further considered [28,29,30]. Following this scoring method, we identified 41 scientific papers that met the established criteria.

In the concluding phase of our literature review, we excluded 6 articles with no full-text availability. As a result, 35 articles were identified as valuable contributors to the scholarly underpinning of our study, as indicated in Figure 1 and Appendix B.

We also included 27 additional supplementary articles, which were freely found during our independent literature search. Despite having lower PEDro scores, all the articles were ISI-indexed, enriching our research further. Thus, the total number of relevant articles for conducting this literature review was 62.

This study was registered in PROSPERO, the international prospective register of systematic reviews, under registration number 575,786.

### 2.2. Funnel Plot Analysis

This funnel plot analysis provides a visual representation of the data extracted from the selected articles, offering insights into the precision and potential biases in the reported studies on the impact of COVID-19 on RA patients.

**Figure 2:**
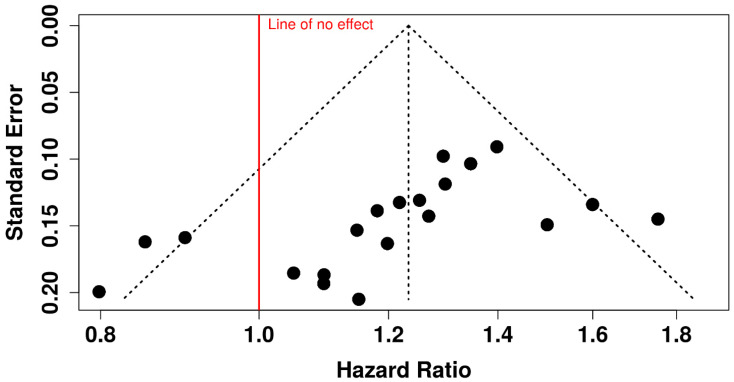
Funnel plot analysis customized for our study.

The funnel plot above (Figure 2) displays the effect sizes, with odds ratio (OR) on the x-axis and the standard error on the y-axis. The black dashed lines represent the 95% confidence intervals, while the solid red line indicates the line of no effect (OR = 1). This visualization helps assess potential publication bias in the analyzed studies.

### 2.3. Funnel Plot Conclusion

The data from the provided studies can be considered accurate based on the credibility of their sources, methodological rigor, thorough peer-review processes, statistical soundness, consistency with existing literature, and adherence to transparency and ethical standards. These factors collectively provide a robust foundation for trusting the data’s accuracy and the validity of conclusions. Further details are presented in Supplementary Material Table S1.

## 3. Results

### 3.1. General Characteristics

Initially, it is essential to underscore the importance of investigating the effects of the pandemic on new RA diagnoses and the accessibility to healthcare.

A study conducted in England noted that the diagnosed cases of RA have increased from 63,781 in 2004 to 107,168 in 2020, with females comprising 70.5% of the total cases. From 2004 to 2012, the yearly incidence of RA exhibited a relatively consistent pattern, fluctuating between 34.5 (95% confidence interval (CI)) and 40 (95% CI) cases per 100,000 person-years. Nevertheless, a significant reduction in RA diagnoses was observed in the year 2020, with the incidence documented at 29.4 (95% CI) per 100,000 person-years [31].

During the early days of the pandemic, many people felt fearful about leaving their homes, even when they were experiencing symptoms that might have necessitated them to seek medical attention [32]. Additionally, due to the isolation measures resorted to by various countries, their access to healthcare was significantly disrupted, leading to delayed diagnoses and treatments [7,31,32]. Telemedicine emerged in this period and was used in a proportion of 30% of patients with an IMID, according to a study that comprised 1517 participants, of which 925 were RA patients. Within this subgroup, 55.7% avoided in-person doctor visits, 40.8% abstained from undergoing laboratory tests, and 28.4% had a telehealth visit [8].

As anticipated, the incidence of RA diagnosis was twice as high in women compared to men, with the peak age of diagnosis occurring in the 65–75-year age group. Similar patterns in annual incidence rates were observed in both males and females across different age groups, except for higher incidence rates in males aged 75 years and older compared to those in the 55- to 65-year age group, while the opposite trend was observed in females [31].

The research mentioned that the prevalence of RA increased by 42.5% between 2004 and 2020, with the annual point-prevalence of RA diagnoses steadily increasing, from 0.541% (95% CI) in 2004 to 0.779% (95% CI) by 2019. In 2020, the percentages of those aged ≥65 to <75 years and ≥75 years with a diagnosis of RA were 1.845% (95% CI) and 2.231% (95% CI), respectively [31].

In 2022, data from the Romanian Registry of Rheumatic Diseases (RRRD) indicated a substantial number of 5396 patients with RA, constituting more than half of the patients with an IMID in the Registry. A typical patient in this cohort would be a female, aged 59.8 years, with a higher body weight, not smoking, living in an urban area, having a high school education, and being actively employed, with an average disease duration of 13 years [33]. It is important to note that the RRRD only includes patients receiving biological treatment. Therefore, this number may not accurately reflect the total number of RA patients in the country. The above results emphasize the need to better characterize RA cohorts in our country, mainly concerning pulmonary infections, most notably COVID-19.

### 3.2. COVID-19 Clinical Manifestations—Acute and Prolonged Symptoms

In a study involving 177 RA patients who contracted SARS-CoV-2, the predominant symptoms observed were fever (15%), cough (12.5%), gastrointestinal symptoms (11.2%), dyspnea (8.8%), neurological symptoms (5%), and cutaneous manifestations (3.8%). In this study, only 15.9% required prolonged hospitalization, and 6.2% succumbed to COVID-19 [9]. In a separate research study involving 128 patients diagnosed with both RA and COVID-19, the predominant symptoms reported were malaise (82.8%), myalgia (82.8%), cough (71.9%), fever (66.4%), anosmia (59.4%), dyspnea (56.3%), ageusia (52.3%), and sore throat (38.3%). This study documented a higher incidence of severe cases, with 38.3% of patients requiring hospitalization, 10.9% necessitating intensive care unit (ICU) support, and 8.6% passing away [4].

Among 3028 patients diagnosed with an IMID across six European countries, 891 of whom were diagnosed with RA, a comprehensive research study concluded that higher concentrations of C-reactive protein (CRP) (OR 1.18, 95% CI; *p* = 0.0063) and a higher number of recent disease flares (OR 1.27, *p* = 0.030) were associated with an increased risk of symptomatic COVID-19. Conversely, the use of biological therapy was found to be associated with a reduced risk [34].

A specific area of intense study pertains to prolonged symptoms, defined as particular manifestations of COVID-19 lasting over 28 days. These symptoms can be further classified as short-term symptoms (one month after the infection), intermediate-term (2–5 months), and long-term (more than 6 months) [35]. A particular study reported that of 174 patients diagnosed with an IMID and COVID-19, 54 experienced prolonged symptoms. The most prevalent manifestations included fatigue (27%), anosmia (18%), dysgeusia (17%), dyspnea (12%), and nasal congestion (12%). Of these patients, 72% did not need to be hospitalized. This research did not address RA specifically [10].

The COVID-19 Global Research Alliance (GRA) Vaccine Survey, which spanned from April to October 2021, encompassed 174 RA patients, of which 32.7% reported prolonged symptoms. Factors correlated with this instance were hospitalization due to COVID-19, mild symptoms (age-adjusted odds ratio (aOR) 6.49, 95% CI), comorbidity count (aOR 1.11 per comorbidity, 95% CI), and the presence of osteoarthritis (aOR 2.11, 95% CI) [11].

### 3.3. COVID-19 Outcomes in Patients with RA

#### 3.3.1. Comorbidities as a Risk Factor for Severe and Critical COVID-19

In our research, we aimed to evaluate prognostic factors associated with severe COVID-19 and mortality among RA patients. Existing literature has offered inconclusive evidence. For example, a study involving 38 RA patients indicated a higher mortality rate from COVID-19 among this group (OR = 2.69, 95% CI) than in the general population, regardless of any risk factor [6].

Another large study undertaken in Greece enrolled 5569 RA patients diagnosed with SARS-CoV-2 infection between 1 January and 30 June 2022 and matched (1:5) on age, sex, and region of domicile random general population comparators. Among these patients, 8.78% required hospitalization, and 1.9% died. Interestingly, there was a higher percentage of SARS-CoV-2-associated hospitalization and deaths in the RA patients compared to the matched controls [18].

In a subgroup analysis from a study comprising 40,014 patients with RA and COVID-19 from Greece in the period 1 March 2020 to 28 February 2021, findings showed an elevated risk of COVID-19-related hospitalization and mortality compared to their counterparts from the general population without any IMID. In comparison to 2019, the overall mortality rate among RA patients in the early stages of the pandemic in Greece surpassed that of the general population (95% CI) [5].

Two Romanian authors conducted a PRISMA systematic literature review regarding the therapeutic management of RA patients. In their assessment, the authors concluded that the progression of RA in patients infected with SARS-CoV-2 is akin to that of the general population, based on data from cross-sectional and cohort studies published thus far. Additionally, they observed increased serum anti-citrullinated protein antibody (ACPA) levels during the infection [2].

Data collected from the Danish National Biobank (DANBIO) Registry, which comprised patients with various IMIDs, of which 8168 patients (71%) had RA, showed that the most prevalent comorbidity was arterial hypertension (HTN) (34%), followed by lung disease and asthma (12%), heart disease (11%), obesity (9%), and DM (7%). It is noteworthy that 26% had two or more associated comorbidities [7]. Depression is highly frequent in RA patients, as indicated by a study involving 400 Romanian patients with RA, which reported a prevalence of 18.8%. Furthermore, depression is acknowledged as a contributing factor to worsening RA symptoms, resulting in increased disease activity. This finding highlights the importance of psychological assistance during the pandemic [36].

RA patients have an increased risk for cardiovascular comorbidities and DM (considering the prolonged inflammation that leads to endothelial damage), but also for certain types of neoplasia [11,37,38]. Neoplasms most commonly associated with RA include those exhibiting a female predominance and furthermore affecting fertility, such as cervical, breast, and ovarian neoplasms, as well as lymphoma, melanoma, and lung cancer. Cervical neoplasia represents a serious health challenge in Romania, with the country ranking second in Europe for the incidence and mortality rates for this condition. This problem is primarily attributed to patients deferring regular screenings due to time and financial constraints. Prioritizing screening for cervical neoplasia in patients with RA is essential [39,40,41].

Comorbidities contribute substantially to the risk of prolonged symptoms [11], hospitalization, and death—this risk is multiplied in patients treated with high-dose corticosteroids and RTX [12,13,14,38,42,43].

The results of a study in Argentina involving 1915 patients with IMIDs, of whom 42% were diagnosed with RA, showed that patients with comorbidities (particularly obesity and pulmonary disease) were more likely to be hospitalized for COVID-19. The likelihood of requiring severe oxygen therapy increased with the presence of two or more comorbidities. Likewise, DM and chronic renal disease were linked to decreased peripheral capillary oxygen saturation (SpO2) and death, while CVD was associated with death [12].

A retrospective cohort study conducted in Canada investigated the influence of comorbidities on the severity and mortality of COVID-19 patients. The research included 167,500 individuals diagnosed with COVID-19 between 15 January and 31 December 2020, and revealed that the presence of RA was significantly associated with increased odds of COVID-19 severity (OR = 1.25, 95% CI 1.09–1.43; *p* < 0.001). Furthermore, the study highlighted that comorbidities were associated with a higher risk of mortality and severity even in individuals under 50 years old, with the presence of ≥5 comorbidities increasing the risk of mortality almost 400 times [13].

The univariate data analysis from a separate study involving 524 patients diagnosed with both RA and COVID-19 indicated a poor prognosis among male patients, individuals over the age of 65, those with active RA, those having comorbidities like HTN, pulmonary disease, and DM, and those undergoing Prednisone (PDN) treatment [44].

DM is well documented in the medical literature and current clinical practice to be linked with worse COVID-19 outcomes, including a higher likelihood of hospitalization and death [42,45], particularly among patients with RA. This association is especially pronounced in individuals using more than 5 mg/day equivalent of PDN. This increased risk is attributed to elevated levels of proinflammatory cytokines, reactive oxygen species, and renin–angiotensin system activation. All these interactions may result in endothelial damage, leading to an increased risk of thromboembolic events, acute lung injury, and lung fibrosis [42,45,46].

More recently, a new perspective on DM suggests that it may have autoimmune origins. This theory proposes that the dysregulated production of proinflammatory adipokines by adipose tissue, influenced by factors like obesity, unhealthy lifestyle, and aging, may lead to insulin resistance and the development of DM. Insulin resistance in patients with RA may be attributed to the increase in proinflammatory cytokines like TNF-α and IL-6 and the extensive use of GCs during the pandemic, especially for patients with low SpO2 levels [42].

#### 3.3.2. The RA Duration as a Determinant of Worse COVID-19 Outcomes

In our literature review, we identified a study carried out at a tertiary center in Ireland from March 2020 to November 2021 that recruited 58 patients with concomitant RA and COVID-19. The 19 patients who succumbed to SARS-CoV-2 exhibited a longer duration of RA (18.8 ± 8.8 years compared to 14.6 ± 6.9 years in the surviving group), showcasing that the duration of RA can also impact the severity of COVID-19 [14].

#### 3.3.3. Immunosuppressive Therapies and Their Intricate Impact on SARS-CoV-2 Infection

In the early stages of the pandemic (1 January–10 June 2020), data from the WHO pharmacovigilance database provided insights into patients with IMIDs and their use of DMARDs. Specifically for the 177 patients with RA, the data revealed the following: 80.1% were using TNF-α inhibitors, with Etanercept (ETA) (41.5%) and Adalimumab (31.2%) being the most commonly prescribed. Tofacitinib and Baricitinib were used by 7.4% of patients, while conventional synthetic disease-modifying anti-rheumatic drugs (csDMARDs) like MTX (8.5%) and Leflunomide (1.1%) were less frequent [9].

A subgroup analysis from a study that involved 925 RA patients revealed that 13,2% had stopped their pathogenic medication with or without a physician’s approval. Peculiarly, from the entire IMID cohort, almost 15% of patients discontinued their DMARD, regardless of whether they had had no respiratory diseases or COVID-19 diagnosis at all. Among patients with respiratory illness, DMARD interruptions were observed in 27.9% of cases, and only 56.4% of these interruptions were recommended by a physician. The study also found that more frequent discontinuation of DMARDs was associated with bDMARDs or Janus kinase inhibitors (JAKis), lower education levels, lower or higher median household income, avoidance of face-to-face consultations, and heightened concerns about COVID-19 [8].

A recent study examined COVID-19 risk factors, symptoms, and prognosis in 128 patients with RA compared to 760 RA patients without COVID-19. According to the multiple logistic regression analysis, the statistically significant variables that were associated with worse COVID-19 outcomes include female gender, obesity, pulmonary disease, chronic kidney disease, PDN ≥5 mg/day, and use of TNF-α inhibitors (95% CI, *p* < 0.05). This study also demonstrated a higher rate of hospitalization among RA patients aged 65 or older, with DM, and individuals receiving treatment with PDN >5 mg/day [4].

(a) Glucocorticoids

The association between corticosteroids and RA bears semblance to the tale of Romeo and Juliet—a fervent liaison marked by the swift and efficacious mitigation of inflammation. However, it is a proscribed relationship, as patients are precluded from their daily use owing to the potent adverse effects that may culminate in tragic consequences.

Nonetheless, corticosteroids were administered in COVID-19 management because of their capability to reduce pulmonary inflammation and enhance oxygenation. It is also a double-edged sword because GCs have been shown to suppress host defense as well as delay microbial clearance, further compromising patients’ susceptibility to serious infections. In contrast, this therapy was proven to reduce the risk of complications in patients with SARS-CoV-2 infection, mainly acute lung injury and the progression to acute respiratory distress syndrome (ARDS) [15].

Furthermore, some studies show that there is an increased risk of mortality in patients who were treated with high-dose GCs. However, this link cannot be attributable solely to the treatment but also to other risk factors [15].

In a study conducted from 1 March 2021 to 1 January 2022, 174 individuals who had recovered from COVID-19 were examined, with 50% of them diagnosed with RA. The study revealed that 18% of the participants reported using GCs at the onset of COVID-19, with a median daily PDN equivalent of 9.5 mg. Moreover, 127 patients were prescribed DMARDs. High-dose corticosteroids were associated with prolonged symptoms, and no mention of SARS-CoV-2 severity was made. However, the study encompassed a broader cohort beyond patients with RA [10].

(b) The interplay of DMARDs and COVID-19

Baricitinib, a JAK1/JAK2 inhibitor, has significantly impacted the recent advancements in RA treatment, including demonstrating superiority over other DMARDs regarding residual pain [47]. It has also been proposed as a potential treatment for COVID-19 due to its role in complement hyperactivation of respiratory epithelial cells and the activation of various immune components that promote senescence of SARS-CoV-2-infected cells, ultimately exacerbating inflammation. Current research indicates that Baricitinib treatment in the context of SARS-CoV-2 infection may lead to clinical and radiologic recovery, a rapid decline in viral load, reduced inflammatory markers, lower COVID-19 mortality rates, and decreased ICU admissions. However, it is crucial to consider its potential risk of co-infections, cardiovascular toxicity (especially thromboembolic events), and adverse hepatic effects [2].

In contrast, treatment with JAKis (not specifically Baricitinib) increased hospitalization risk (OR: 2.18; 95% CI) in a large Greek cohort study [18].

The severity of SARS-CoV-2 infection can be attributed in part to the cytokine storm, primarily driven by compromised interferon and cytokine responses (TNF-α, IL-1, IL-6), endothelial activation, inflammation, and immunothrombosis [2,9,19]. Based on the aforementioned points, it is reasonable to speculate that inhibiting these cytokines could substantially reduce mortality rates. Additionally, this treatment is already available within the scope of RA therapeutics.

Despite the capacity of this therapy to increase the risk of infections [9], patients undergoing treatment with TNF-α inhibitors exhibit a reduced susceptibility to adverse outcomes associated with COVID-19 in contrast to those receiving GCs. As per the guidelines established by the European League Against Rheumatism (EULAR) and the American College of Rheumatology (ACR), it is advised that individuals with RA should not discontinue or diminish their treatment regimen during the COVID-19 pandemic. Furthermore, RA patients who are prone to experiencing disease exacerbation, even with brief interruptions in medication, should have access to an adequate supply of IL-1, IL-6, and JAK inhibitors [19].

TCZ, a monoclonal antibody targeting IL-6 receptors, has demonstrated early promise in managing the COVID-19 pandemic. This treatment has shown a 5% increase in oxygenation efficacy, improvement in “ground-glass” opacities seen on pulmonary computer tomography, a reduction in the risk of mechanical ventilation, and a 95% increase in hospital discharge rates. However, specific trials have indicated that TCZ may not confer additional benefits in preventing intubation or mortality in hospitalized COVID-19 patients with severe pneumonia. Adverse effects associated with TCZ include transient liver injury, hypertriglyceridemia, acute pancreatitis, and pancytopenia [2,19].

Rituximab is an immunosuppressive agent (a chimeric monoclonal antibody against the cluster of differentiation-20 (CD-20)) utilized in managing a range of malignancies and rheumatologic disorders, including RA. However, the utilization of RTX can lead to swift B-cell depletion, resulting in secondary hypogammaglobulinemia, compromised opsonization, and an inability to mount an antibody response to exposed antigens. The recovery from this condition may extend up to 12 months. Consequently, these individuals become predisposed to infections and associated complications, thereby elucidating the increased severity of COVID-19 in immunocompromised patients treated with RTX [14,19].

In a study conducted from 2020 to 2021 involving Romanian patients suffering from an IMID, 298 individuals who contracted SARS-CoV-2 were examined. Among this cohort, 53.69% were diagnosed with RA. Among those with severe cases, 26.19% received treatment with RTX, 42.85% received treatment with TNF-α inhibitors, 16.66% were treated with JAKis, 9.52% received TCZ, 2.38% were given secukinumab, and 2.38% were prescribed ABA. Only 4.02% of patients succumbed to the illness and were undergoing treatment with RTX, tofacitinib, ETA, secukinumab, and TCZ [17].

In another study on 5569 RA patients diagnosed with COVID-19, it was shown that RTX treatment conferred an increased risk for hospitalization (OR: 6.12; 95% CI) and death (OR: 12.06; 95% CI) [18].

A large study in the United States (US) assessed the impact of RTX treatment on patients with RA and COVID-19 in the period 1 January 2020–16 September 2021. A total of 22,956 RA patients were diagnosed with COVID-19. As treatment for RA, 60.27% of patients received GCs, 45.53% were treated with csDMARDs, 5.51% were prescribed JAKis, 14.6% received TNF-α inhibitors, 2.68% were administered IL-6 inhibitors, 2.25% received RTX, and 2.92% were treated with ABA. Another important finding was that 18.38% of patients required hospitalization, 0.84% necessitated admission to the ICU, and 4.7% were deceased. In the context of multivariate-adjusted models, it was ascertained that relative to the baseline usage of csDMARDs, the administration of RTX exhibited an important association with COVID-19-related hospitalization (aOR:2.14, 95% CI). Moreover, among hospitalized individuals, the utilization of RTX was concomitantly associated with increased probabilities of ICU admission (aOR 5.22, 95% CI) and invasive ventilation (aOR 2.74, 95% CI). Of particular note, individuals who had undergone RTX infusions more than 180 days earlier exhibited diminished 30-day mortality rates (OR:0.25, 95% CI). However, no statistically significant difference in 30-day mortality rates was discerned among those who had received RTX infusions between 31 and 180 days prior [16].

With respect to other therapies, a recent study conducted in the United States and Spain shows that non-invasive vagus nerve stimulation (nVNS) may have potential for the treatment of the excessive inflammation response seen in COVID-19 patients. The study also showed that patients undergoing nVNS therapy had decreased levels of inflammatory markers such as CRP and procalcitonin. From this report, it can be inferred that nVNS can be another therapeutic option in the management of the inflammatory cascade generated by COVID-19 [48].

### 3.4. Vaccination against SARS-CoV-2 in Patients with RA: Insights into Humoral and Cellular Immunity Responses

#### 3.4.1. General Aspects

Vaccination against COVID-19 has proven to be an important instrument tool in combating the pandemic. Immunization was an opportunity and a challenge at the same time for any patient with RA. The use of COVID-19 vaccines in immunocompromised individuals has been under massive study and much controversy, especially since patients with IMIDs were notably excluded from the initial vaccine clinical trials [2]. These people might have certain concerns regarding how the underlying disease or immunomodulating therapies influence these vaccines’ effectiveness and/or risk. Moreover, there was a lack of standardization in the distribution/administration and availability of vaccines, which, together with conflicting information from the RA physicians and official recommendations, further complicated these concerns [20,49,50].

Various studies and real-world evidence assessments reveal that the SARS-CoV-2 vaccination is generally safe and effective for RA patients, despite a relatively blunted immune response compared to the general population. Moreover, case series have pointed to a possible worsening of RA symptoms following COVID-19 vaccination, especially for those receiving DMARDs [2,51].

The vaccines listed in our article include BNT162b2, mRNA-1273/CX-024414, ChAdOx1 nCoV-19/AZD1222, JNJ-78436735/Ad.26.COV2.S, NVX-CoV2373, BBIBP-CorV, and BBV152.

Evidence from a US study that enrolled 243 patients diagnosed with an IMID, of whom 121 were diagnosed with RA, acknowledged that attitudes toward vaccination were highly positive. For example, 92% of the participants had taken a flu shot in the previous year, and 84% wanted to receive a COVID-19 vaccine at the earliest opportunity, considering this would avoid primary infection [49].

In a large retrospective study that enrolled 5569 patients diagnosed with both RA and SARS-CoV-2 infection, in Greece, it was observed that various forms of immunity, including previous vaccination, prior infection, and “hybrid immunity” (infection and vaccination), protected the individuals against COVID-19-related hospitalization. These findings demonstrate that previous vaccination without prior infection is protective against COVID-19-associated severity. Additionally, hybrid immunity granted better outcomes, with only 1 death reported among 127 patients with this type of protection [18].

An observational study performed in Denmark from January to October 2021 collected data from RA patients identified in the DANBIO Register and then matched these individuals in a 1:20 ratio from the general population, based on age, gender, and vaccination status. Among the 28,447 unvaccinated individuals with RA, 71.3% were women, with a median age of 67.7 years. The hospitalization rate was 0.2%, identical to that of the matched general population. The most frequent comorbidity observed was CVD (20.6%). With respect to the treatment used, 55.5% were undergoing therapy with MTX, 14.2% with SSZ, 12.5% with PDN, and 16.9% with TNF-α inhibitors. Of the 26,217 fully vaccinated individuals also diagnosed with RA, 71% were women, with a median age of 68.9. The hospitalization rate was 0.4%, similar to that of the matched general population and unvaccinated RA patients. The most frequent comorbidity observed was CVD (21.7%). Additionally, 55.7% were treated with MTX, 13.5% with SSZ, 12.2% with PDN, and 17.1% with TNF-α inhibitors. Furthermore, 89.2% of the individuals were immunized with BNT162b2 and 8.4% with mRNA-1273 [52].

#### 3.4.2. RA and Impaired Vaccine Response

Research shows that the reduced effectiveness of the BNT162b2 vaccine is mainly due to the dysregulated immune system of RA patients, which results from the disease itself and immunosuppressive therapies like MTX and RTX [52,53].

It is imperative to underline that the immunogenicity should not only be measured by the antibody titers but also include an assessment of T-cell responses and interferon (IFN) activity [53].

A prospective study was conducted in the United Kingdom (UK), in 2021, to assess 100 patients with RA who tested positive for ACPA and received either BNT162b2 or ChAdOx1 nCoV-19/AZD1222 vaccines. The study’s objective was to analyze antibody and T-cell activity (measured by IFN I activity) before and four weeks after vaccination. Patients who did not have any antibody response after the first dose of the vaccine were retested four weeks after the second dose and then after the third one, if they remained seronegative. In individuals who had not been infected prior to vaccination, 45% developed antibodies, 53% manifested T-cell responses, and 77% demonstrated either antibody or T-cell responses following a single dose of vaccine. Furthermore, 19 additional patients (54%) subsequently demonstrated seroconversion following a second vaccine dose, and only 2 more individuals exhibited vaccine response after a third dose. In summary, 11% (8/74) of patients failed to seroconvert following three vaccine doses [53].

#### 3.4.3. Specific DMARDs and Impaired Vaccine Response

While proving effective in managing RA flares, RTX therapy has considerable impacts on the ability of a patient’s body to mount a humoral response to COVID-19 vaccination. Nonetheless, there is an established observation that T-cell-mediated immunity is normal in the overwhelming majority of patients with IMIDs despite the diminished humoral response [54].

In the aforementioned study conducted in the UK [53], no patients treated with ABA developed seroconversion, as compared to the other four treatment groups: RTX (35% of patients developed immunity), anti-TNF-α (65%), JAKis (56%), and anti-IL-6 (63%). Conversely, all healthy controls (HCs) exhibited antibody responses (*p* < 0.001). Among patients treated with RTX for over six months from their initial SARS-CoV-2 vaccination, 57% exhibited detectable antibody responses, compared to 13% of those treated for less than six months (OR: 2; 95% CI; *p* = 0.012). Reduced seroconversion rates were observed in patients concurrently using MTX. There were no discernible differences in SARS-CoV-2 patients’ T-cell response rates between different DMARDs or using concomitant MTX [53]. Factors associated with seroconversion after a single vaccine dose included age ≤50 years old, (OR: 18, 95% CI, *p* = 0.012), six months or more from the last RTX infusion (OR: 10, 95% CI, *p* = 0.029), and anti-TNF-α treatment compared with RTX (OR: 12, 95% CI, *p* = 0.012). Conversely, factors associated with failure to develop antibody responses included absent previous infection (OR: 0.01, 95% CI, *p* ≤ 0.001) and concomitant MTX usage (OR: 8, 95% CI, *p* = 0.01). Moreover, a trend for better T-cell responses was observed in patients treated with JAKis (OR: 0.24, 95% CI, *p* = 0.079) and ABA (OR: 0.26, 95% CI, *p* = 0.098) compared to RTX, as well as in those who had not received recent GCs (OR: 0.28, 95% CI, *p* = 0.083) [53].

A recent study that enrolled 90 RA patients undergoing treatment with RTX and 1114 matched controls assessed the humoral and cellular responses to two and three doses of the BNT162b2, mRNA-1273, and ChAdOx1 nCoV-19 vaccines. The findings revealed that only 21.8% of RA patients, as opposed to 98.4% of HCs, exhibited a serological response after receiving two vaccine doses. Furthermore, adding a third dose did not significantly improve these numbers, with only 16.3% of 49 patients showing a serological response. The median interval between the last RTX infusion and the third vaccine dose was 250 days. With respect to T-cell response, the study observed that after two vaccine doses, 53% of patients developed specific CD4+ T-cell responses, and 74% displayed specific CD8+ T-cell responses, with no specific correlation to the time elapsed since the last RTX infusion. Conversely, all HCs demonstrated T-cell response. After the administration of the third vaccine dose, all RA patients exhibited a T-cell response [22].

MTX has been identified as potentially affecting the body’s response to COVID-19 and influenza vaccines. This is attributed to its capacity to reduce humoral immune responses by interacting with the B-cell activation factor, generating immunoregulatory adenosine, and inducing regulatory B cells’ activation [55,56].

A study revealed that temporarily pausing MTX for 14 days after receiving a COVID-19 booster shot (in patients with any IMID, with 51% having RA) resulted in a 2.19-fold increase in antibody response after four weeks, and this enhanced response was sustained at 12 weeks. These findings were consistent across different demographic groups and MTX doses [55].

Additionally, a different study also indicated that discontinuing MTX during the second dose of the ChAdOx1 nCoV-19 vaccine may be a safe and effective approach to augmenting antibody response in patients with RA [56].

A recent study involving 35 patients examined the effects of the BNT162b2-mRNA vaccine on individuals with RA undergoing treatment with various DMARDs. The study found that 97% of the patients exhibited specific humoral immunity. However, compared to HCs, the antibody titer was lower in patients receiving cytotoxic T-lymphocyte-associated antigen 4 (CTLA-4) inhibitors (ABA) and IL-6 inhibitors (*p* < 0.001). Moreover, a positive T-cell-specific response was observed in the majority of RA patients (69%). Individuals undergoing certain biological therapy (IL-6 and TNF-α inhibitors, ABA) had significantly lower IFN levels than HCs [22].

Another study carried out a comparative analysis between 35 RA patients and 49 healthcare workers (HCWs) without any IMID. The findings revealed reduced neutralizing IgG titers over time in both HCWs and RA patients after the administration of the BNT162b2 vaccine. In contrast to HCWs, RA patients undergoing TNF-α inhibitor and ABA treatment developed significantly lower median antibody titers at six months. Qualitative and quantitative IFN responses were diminished among RA patients at the 6-month mark. A subset of RA patients developed a T-cell-specific response at six months, a phenomenon that was absent at the five-week assessment. Furthermore, there was an increase in T-cell response among 41.4% of RA patients compared to 26.2% observed in HCWs at the six-month post-vaccination period. These results suggest a potential delayed cellular immunity in RA patients [57].

The diminished antibody response to the COVID-19 vaccines is attributed to the well-documented impact of the CTLA-4-Ig and TNF-α inhibitors on reducing the frequency of peripheral memory B cells in humans’ peripheral blood [57].

#### 3.4.4. Safety Concerns

Inactivated COVID-19 vaccination is considered safe for RA patients, although some of them may experience Varicella Zoster Virus (VZV) reactivation [57,58,59].

An analysis of a subgroup from a study involving 101 RA patients, of which 92% received the BNT162b2 vaccine and 8% received the mRNA-1273 vaccine, showed that 55.4% reported no adverse effects. The remaining participants reported experiencing pain at the injection site (33%), headache (almost 9%), fever (approximately 8%), and fatigue (5%) [60].

A recent study involving 128 systemic lupus erythematosus (SLE) and 154 RA patients who received the BNT162b2 vaccine acknowledged that the majority of adverse reactions were mild, i.e., primarily local reactogenicity (78.0%). The most severe category of AEs (categorized as grade 4—defined as instances requiring an emergency department visit or hospitalization, necrosis or exfoliative dermatitis, or fever above 40 °C) occurred in less than 1% of patients in this group [23].

In a subgroup analysis of 2427 patients with RA within 21 days post-vaccination, the adjusted incidence rate ratio (aIRR) for an AIRD (IMID o.n.) flare was 0.85 (95% CI), suggesting that vaccination was not linked to disease flares in this patient population [59].

An important question that emerges regarding adverse events of vaccination is related to rheumatological symptoms and new-onset undifferentiated arthritis (UA) or even newly diagnosed RA. A recent original article [61] demonstrated that from 109 patients who had rheumatological complications post-COVID-19 (PC) and post-COVID-19 vaccine (PCV), 87 had UA, and 22 were diagnosed with Polymyalgia Rheumatica and Horton’s arteritis. In UA cases, 43 PC and 44 PCV were observed. Furthermore, positivity for rheumatoid factor was found in 11.5% of cases and for ACPA in 5.7% of individuals. Moreover, the ultrasound evaluation revealed power-Doppler (PD)-active synovitis, proliferative tenosynovitis, and pseudo-tenosynovitis in 85.1%, 65.5%, and 60.9% of individuals, respectively. Furthermore, bone erosions—a hallmark of early RA—were found in 5.7% of patients. At the 3-month evaluation, “75.9% of patients met the ACR/EULAR 2010 criteria for early arthritis”, thus suggesting the potential for this virus and its vaccine to induce RA [61].

From a study performed in Switzerland that enrolled 1347 RA patients and interviewed them regarding their vaccination experiences and vaccine-related events, we found out that the majority (76.9%) reported minor AEs. A small percentage (4.2%) experienced major AEs, with three patients (0.2%) needing hospitalization. The most frequently reported minor AEs included pain at the injection site (59.2%), fatigue (27.2%), headache (25.6%), and myalgias (22.7%). Reported specific major adverse events included anaphylaxis (0.1%), dyspnea (0.5%), and severe skin rashes (0.6%) [24].

Vaccine-related AEs and hospitalization rates did not differ significantly between patients with active and inactive RA, except for a higher incidence of dizziness in those with active RA (OR = 2.1; 95%CI; *p* = 0.021). Major AEs were consistent across different vaccine recipients, except for individuals who received the JNJ-78436735 vaccine, who reported a higher frequency of major AEs compared to the rest of the population (OR = 17.0; 95%CI; *p* < 0.001). The incidence of hospitalizations subsequent to vaccination was uncommon and exhibited consistency across various vaccinated RA individuals. Thus, recipients of the ChAdOx1 nCoV-19 and mRNA-1273 vaccines reported systemic minor AEs with higher frequency than patients immunized with BNT162b2, BBV152, and BBIBP-CorV. Minor gastrointestinal AEs were more frequently reported by recipients of the ChAdOx1 nCoV-19 (OR = 2.1; 95%CI; *p* = 0.003) and mRNA-1273 (OR = 3.4; 95%CI; *p* < 0.001) vaccines. Other adverse events following vaccination (tachycardia, elevated blood pressure, thoracic discomfort, and vertigo) have been infrequently reported [24].

Upon cessation of MTX two weeks following each vaccine administration, a study conducted in India revealed that patients experienced an elevated frequency of fever, myalgia, and headache [56].

Patients that were treated with MTX experienced significantly reduced rates of tachycardia (OR = 0.3; 95%CI; *p* = 0.002), breathing difficulties (OR 0.15, 95%CI, *p* = 0.002), vertigo (adjusted OR = 0.6, 95%CI, *p* = 0.022), and thoracic discomfort (aOR = 0.3, 95%CI, *p* = 0.026). On the other hand, individuals receiving HCQ more frequently reported bodily discomfort (aOR = 1.3, 95%CI, *p* = 0.028) and less frequently reported headaches (aOR 0.7, 95% CI, *p* = 0.024), fatigue (aOR = 0.5, 95%CI, *p* < 0.001), and vertigo (aOR = 0.5, 95%CI, *p* = 0.020). AEs related to vaccination showed no significant difference among individuals receiving different forms of immunosuppression, and hospitalization rates were comparable across the groups [24].

#### 3.4.5. Breakthrough Infections in Individuals with RA

A vaccine breakthrough infection with SARS-CoV-2 refers to the detection of the virus in a respiratory (e.g., nasal, throat/nasopharyngeal) specimen collected from a person ≥14 days after receipt of all recommended doses; this does not encompass reinfections involving new exposure [54].

In a study conducted in the UK, 400 patients with IMIDs were included, 272 of whom were diagnosed with RA, all undergoing treatment with RTX. Among the RA group, the median number of previous RTX cycles of administration was six, with 60% concurrently using MTX and 15% using low-dose PDN. Additionally, 4% of the patients exhibited hypogammaglobulinemia. Moreover, 370 patients with IMID who had been administered at least two vaccine shots were included in the study. Within this group, 110 (30%) had breakthrough infections; among these, 16 had moderate-to-severe COVID-19, and one death was reported. The mean time to COVID-19 of any severity and moderate–severe infection after the second vaccine dose was 32.2 weeks (SD: 12.2) and 28.5 weeks (SD: 12.4), respectively. The study did not report specific information about RA-related breakthrough cases. In the multivariate analysis, the presence of comorbidities (hazard ratio (HR): 1.46; 95% CI; *p* = 0.0037) and low IgG concentrations before RTX treatment were associated with higher risk for moderate-to-severe COVID-19 outcomes (*p* = 0.014). The risk decreased with each vaccine dose received (OR 0.49; *p* < 0.0001) [54].

In a separate study, it was found that fully vaccinated RA patients receiving RTX may have an increased risk of breakthrough COVID-19 infections and subsequent hospitalization [16]. Of note, no consistent pattern was observed with regard to the duration between the last RTX administration and vaccination or hospitalization.

## 4. Discussion

The results of our PRISMA systematic literature review offer important insights into the complex relationship between RA and COVID-19. Our findings underscored challenges in the area of immunological issues, diagnosis, treatment (including its accessibility), vaccination, and severity of COVID-19 outcomes in this vulnerable population. These results are consistent with literature data that indicate an increased risk of severe SARS-CoV-2 in RA patients due to their immunocompromised status caused by their underlying condition and the immunosuppressive therapies they undergo [4,5,6,16,18].

One striking observation is the marked decline in RA diagnoses in 2020, probably influenced by pandemic-induced hesitancy regarding approaching healthcare services and disruption to health services in general [7,8]. This reduction in the number of diagnoses draws attention to the need for accessible healthcare even during global crises and the enhancement of patients’ trust in doctors, since delayed diagnosis of RA is associated with worse long-term outcomes.

The symptomatology linked to COVID-19 showed a very complex mutual interaction between the two conditions. The presence of fever, cough, dyspnea, and gastrointestinal manifestations was consistent across various studies, although the incidence and severity varied [4,9,38]. Another important mention is that prolonged symptoms after infection with SARS-CoV-2, including fatigue, dyspnea, and musculoskeletal pain, are more frequent in the RA population and need a careful differential diagnosis and approach to treatment [10,11].

Comorbidities are a determinant factor related to the severity of COVID-19 among RA patients. The presence of conditions such as HTN, obesity, DM, and CVD increased the risks of hospitalization, severe disease, and mortality. These findings agree with the general literature indicating that certain comorbidities exacerbate COVID-19 severity, especially in a population that was already experiencing chronic inflammatory diseases like RA [12,13,14,38,42,43,44].

Furthermore, our study identified a significant association of disease duration of RA with COVID-19 infection outcomes; long-standing RA may thus be another factor contributing to poor prognosis in such infected patients [14]. This association can be attributed to the persistent immune inflammation that hampers the host’s defense capacity.

The fragile balance of immunosuppressive therapies in the setting of COVID-19 for RA patients creates a complex landscape and requires a nuanced understanding of immunomodulation, infection risk, and therapeutic outcomes. GCs, for instance, while effective in controlling RA inflammation, are a double-edged sword in the context of COVID-19. Their immune-suppressing effects on pulmonary inflammation are beneficial on one hand, but on the other hand, this simultaneously increases vulnerability to opportunistic infections, especially when they are administered in high doses. Nonetheless, they have been shown to reduce the risk of severe complications such as ARDS in COVID-19, demonstrating the delicate balance required in their administration [4,10,15].

DMARDs further complicate this landscape. For example, drugs such as Baricitinib and Tocilizumab have been found to decrease the severity of COVID-19, improve clinical outcomes, and reduce mortality rates. Similarly, TNF-α inhibitors can reduce the severity of SARS-CoV-2 infection by controlling the well-known related cytokine storm, but their use increases the general risk of infections [2,9,10,19].

In contrast, another important bDMARD, RTX, reduced the defense capacity of RA patients, according to the literature data [2,14,16,17,18]. However, it is essential to underline that additional factors like RA duration and severity, and thus prolonged systemic inflammation, may interfere with this relationship because RTX is often a second-line therapy for patients with refractory RA [38,62].

Our study has also determined and highlighted the role of vaccination in protecting RA patients against severe COVID-19 outcomes. However, vaccine efficacy, considering both humoral and cellular responses, is often compromised by the underlying autoimmune disorder and the immunosuppressive treatments employed [20,22,60]. Studies indicate that vaccination is generally safe and effective for RA patients, with a low rate of disease flares [23,24,59,60,63,64] and potential new-onset RA [61,62] following vaccination. However, treatment with MTX [53,55,56,65], ABA [22,53,57,65], TNF-α inhibitors [21,22,57,66], and RTX [53,54] may impair the immune response after vaccination. This issue necessitates personalized vaccination strategies, including the potential for booster doses and timing adjustments to optimize immunogenicity.

It is important to underline a specific aspect regarding COVID-19 in RA patients: the potential to trigger other autoimmune diseases, for instance, Hashimoto’s Disease. Although no clear evidence exists for such an association, to our knowledge, the intimate molecular mechanisms supporting this potential problem are obvious. For instance, the cytokine storm present in the severe forms of COVID-19 can exacerbate the already altered immune response in RA patients, thus potentially triggering other autoimmune diseases. Moreover, the activation of Toll-like receptors (TLRs) by SARS-CoV-2 (especially TLR 7, which is also linked with the genetic susceptibility for IMIDs [2]) might contribute to chronic inflammation and autoimmunity, leading to conditions like autoimmune thyroiditis, type 1 diabetes, or Guillain–Barré syndrome, in predisposed individuals [67,68,69]. It is mandatory not to forget the molecular mimicry between the SARS-CoV-2 proteome and endogenous proteins or antigens, such as thyroid proteins (thyroglobulin and thyroid peroxidase), pancreatic beta-cell antigens, myelin basic protein, heat shock protein 60, and many others that can lead to cross-reactive immune responses targeting other tissues [70,71,72,73].

The limitations of this systematic literature review consist mainly of the rather short targeted area of search for literature-related resources, but this is an objective constraint due to the recent onset of the COVID-19 pandemic. Furthermore, our literature review did not assess the analysis of specific SARS-CoV-2 variants; identifying a specific variant responsible for infection requires genomic sequencing, which is not part of routine screening, and adding this method of diagnosis as an inclusion criterion in studies can limit the number of suitable patients and implicitly the statistical power of the related analysis. This underscores a literature gap and calls for further studies. Another important issue that emerged as a question for subsequent research and was not addressed in the present literature review is the new onset of autoimmune diseases in RA patients following infection and/or vaccination.

Additional Considerations

The available literature on the correlation between COVID-19 and RA needs to be revised. Our study highlights knowledge gaps and underscores the need for further comprehensive research concerning the impact of SARS-CoV-2 infection on this particular group of patients in Romania. This work is part of my PhD research regarding the impact of COVID-19 on Romanian patients with IMID. Further studies will focus on the real-data analysis of such cohorts in our country.

## 5. Conclusions

SARS-CoV-2 infection has undeniably altered the management and outcomes of patients with RA. The decline in RA diagnoses during the pandemic and the increased severity of COVID-19 in RA patients, especially those suffering from certain comorbidities and/or immunologically influenced by DMARD therapy, calls for a more resilient healthcare approach, which would ensure continuity in the care for chronic patients even during times of crisis. The high prevalence of prolonged COVID-19 symptoms among RA patients underscores the necessity for long-term monitoring of this population.

Moreover, the association between long-standing RA and poor COVID-19 outcomes, together with the complex influence of immunosuppressive therapies and vaccination, suggests that personalized treatment strategies are essential in managing RA patients.

Ongoing research and adaptive treatment strategies will, therefore, become of absolute importance in this ever-changing environment, ensuring that RA patients receive comprehensive, individualized care beyond the COVID-19 pandemic. The resilience of patients and healthcare providers in adapting to these challenges will undoubtedly shape a more informed and compassionate healthcare system moving forward.

## Data Availability

Not applicable.

## Supplementary Materials

The following supporting information can be downloaded at https://www.mdpi.com/article/10.3390/ijms252011149/s1.

## Abbreviations

ABA	Abatacept
ACPA	Anti-citrullinated protein antibody
ACR	American College of Rheumatology
aIRR	Adjusted incidence rate ratio
aOR	Adjusted odds ratio
ARDS	Acute respiratory distress syndrome
bDMARD	Biologic disease-modifying antirheumatic drug
CD	Cluster of differentiation
CI	Confidence interval
COVID-19	Coronavirus Disease 2019
CRP	C-reactive protein
CVD	Cardiovascular disease
csDMARD	Conventional synthetic disease-modifying antirheumatic drug
DANBIO	Danish National Biobank
DM	Diabetes mellitus
DMARD	Disease-modifying antirheumatic drug
EULAR	European League Against Rheumatism
ETA	Etanercept
GC	Glucocorticoid
GRA	Global Research Alliance
HCQ	Hydroxychloroquine
HTN	Hypertension
ICU	Intensive care unit
IL	Interleukin
IMID	Immune-mediated inflammatory disease
ISI	Institute for Scientific Information
JAKi	Janus kinase inhibitor
MTX	Methotrexate
NCBI	National Center for Biotechnology Information
nVNS	Non-invasive vagus nerve stimulation
OR	Odds ratio
PC	post-COVID-19
PD	Power Doppler
PCV	post-COVID-19 vaccine
PDN	Prednisone
PMC	PubMed Central
PRISMA	Preferred Reporting Items for Systematic Reviews and Meta-Analyses
RA	Rheumatoid arthritis
RTX	Rituximab
SARS-CoV-2	Severe acute respiratory syndrome coronavirus 2
SD	Standard deviation
SpO_2_	Peripheral capillary oxygen saturation
TCZ	Tocilizumab
TLR	Toll-like Receptor
TNF-α	Tumor necrosis factor-alpha
tsDMARD	Targeted synthetic disease-modifying antirheumatic drug
WHO	World Health Organization
UA	undifferentiated arthritis
UK	United Kingdom
US	United States

## Appendix A

**Column 1**	**Column 2**	**Column 3**	**Column 4**	**Column 5**	**Column 6**
**Start date:**	**1 January 2021**				
**End date:**	**31 December 2023**				
**No of keywords**	**7**				
	**Part 1**	**Part 2**	**Part 3**	**Part 4**	**Part 5**
**Keywords 1**	COVID-19	rheumatoid arthritis	symptoms	hospitalization	flare
**Keywords 2**	COVID-19	RA	symptoms	hospitalization	flare
**Keywords 3**	SARS-CoV-2	rheumatoid arthritis	symptoms	hospitalization	flare
**Keywords 4**	SARS-CoV-2	RA	symptoms	hospitalization	flare
**Keywords 5**	Novel Coronavirus Disease	rheumatoid arthritis	symptoms	hospitalization	flare
**Keywords 6**	Novel Coronavirus Disease	RA	symptoms	hospitalization	flare
**Keywords 7**	COVID-19	rheumatoid arthritis	corticosteroids		
**Keywords 8**	COVID-19	RA	corticosteroids		
**Keywords 9**	SARS-CoV-2	rheumatoid arthritis	corticosteroids		
**Keywords 10**	SARS-CoV-2	RA	corticosteroids		
**Keywords 11**	Novel Coronavirus Disease	rheumatoid arthritis	corticosteroids		
**Keywords 12**	Novel Coronavirus Disease	RA	corticosteroids		
**Keywords 13**	COVID-19	rheumatoid arthritis	DMARD		
**Keywords 14**	COVID-19	RA	DMARD		
**Keywords 15**	SARS-CoV-2	rheumatoid arthritis	DMARD		
**Keywords 16**	SARS-CoV-2	RA	DMARD		
**Keywords 17**	Novel Coronavirus Disease	rheumatoid arthritis	DMARD		
**Keywords 18**	Novel Coronavirus Disease	RA	DMARD		
**Keywords 19**	COVID-19	rheumatoid arthritis	disease-modifying rheumatic drug		
**Keywords 20**	COVID-19	RA	disease-modifying rheumatic drug		
**Keywords 21**	SARS-CoV-2	rheumatoid arthritis	disease-modifying rheumatic drug		
**Keywords 22**	SARS-CoV-2	RA	disease-modifying rheumatic drug		
**Keywords 23**	Novel Coronavirus Disease	rheumatoid arthritis	disease-modifying rheumatic drug		
**Keywords 24**	Novel Coronavirus Disease	RA	disease-modifying rheumatic drug		
**Keywords 25**	COVID-19	rheumatoid arthritis	C Reactive Protein		
**Keywords 26**	COVID-19	RA	C Reactive Protein		
**Keywords 27**	SARS-CoV-2	rheumatoid arthritis	C Reactive Protein		
**Keywords 28**	SARS-CoV-2	RA	C Reactive Protein		
**Keywords 29**	Novel Coronavirus Disease	rheumatoid arthritis	C Reactive Protein		
**Keywords 30**	Novel Coronavirus Disease	RA	C Reactive Protein		
**Keywords 31**	COVID-19	rheumatoid arthritis	chest imaging		
**Keywords 32**	COVID-19	RA	chest imaging		
**Keywords 33**	SARS-CoV-2	rheumatoid arthritis	chest imaging		
**Keywords 34**	SARS-CoV-2	RA	chest imaging		
**Keywords 35**	Novel Coronavirus Disease	rheumatoid arthritis	chest imaging		
**Keywords 36**	Novel Coronavirus Disease	RA	chest imaging		
**Keywords 37**	COVID-19	rheumatoid arthritis	sequelae	admission risk	
**Keywords 38**	COVID-19	RA	sequelae	admission risk	
**Keywords 39**	SARS-CoV-2	rheumatoid arthritis	sequelae	admission risk	
**Keywords 40**	SARS-CoV-2	RA	sequelae	admission risk	
**Keywords 41**	Novel Coronavirus Disease	rheumatoid arthritis	sequelae	admission risk	
**Keywords 42**	Novel Coronavirus Disease	RA	sequelae	admission risk	
**Keywords 43**	COVID-19	rheumatoid arthritis	vaccination	age	
**Keywords 44**	COVID-19	RA	vaccination	age	
**Keywords 45**	SARS-CoV-2	rheumatoid arthritis	vaccination	age	
**Keywords 46**	SARS-CoV-2	RA	vaccination	age	
**Keywords 47**	Novel Coronavirus Disease	rheumatoid arthritis	vaccination	age	
**Keywords 48**	Novel Coronavirus Disease	RA	vaccination	age	

## Appendix B

**No.**	**Article**	**Publ. Year**	**No. of ** **Citations**	**No. of ** **References**	**No. of Citations/Year**	**PEDro Score**
1	Rituximab is associated with worse COVID-19 outcomes in patients with rheumatoid arthritis: A retrospective, nationally sampled cohort study from the U.S. National COVID Cohort Collaborative (N3C)	2023	15	24	15	10
2	Effect of a 2-week interruption in methotrexate treatment versus continued treatment on COVID-19 booster vaccine immunity in adults with inflammatory conditions (VROOM study): a randomised, open label, superiority trial	2022	46	30	23	10
3	Impaired immunogenicity to COVID-19 vaccines in autoimmune systemic diseases. High prevalence of non-response in different patients’ subgroups	2021	80	27	26.66667	10
4	Breakthrough SARS-CoV-2 infections and prediction of moderate-to-severe outcomes during rituximab therapy in patients with rheumatic and musculoskeletal diseases in the UK: a single-centre cohort study	2023	21	34	21	10
5	Humoral and cellular immune responses to two and three doses of SARS-CoV-2 vaccines in rituximab-treated patients with rheumatoid arthritis: a prospective, cohort study	2022	111	29	55.5	10
6	COVID-19 and corticosteroids: a narrative review	2022	35	166	17.5	10
7	Kinetics of the B- and T-Cell Immune Responses After 6 Months From SARS-CoV-2 mRNA Vaccination in Patients With Rheumatoid Arthritis	2022	33	55	16.5	10
8	ImmunosuppressiveTherapies Differently Modulate Humoral- and T-Cell-Specific Responses to COVID-19 mRNA Vaccine in Rheumatoid Arthritis Patients	2021	68	41	22.66667	10
9	Concerns, Healthcare Use, and Treatment Interruptions in Patients With Common Autoimmune Rheumatic Diseases During the COVID-19 Pandemic	2021	54	17	18	10
10	Early experience of COVID-19 vaccination in adults with systemic rheumatic diseases: results from the COVID-19 Global Rheumatology Alliance Vaccine Survey	2021	117	32	39	10
11	Association Between Immune Dysfunction and COVID-19 Breakthrough Infection After SARS-CoV-2 Vaccination in the US	2022	147	34	73.5	10
12	Low frequency of disease flare in patients with rheumatic musculoskeletal diseases who received SARS-CoV-2 mRNA vaccine	2022	30	18	15	10
13	Association of pre-existing comorbidities with mortality and disease severity among 167,500 individuals with COVID-19 in Canada: A population-based cohort study	2021	65	32	21.66667	10
14	Impaired Antibody Response to the BNT162b2 Messenger RNA Coronavirus Disease 2019 Vaccine in Patients With Systemic Lupus Erythematosus and Rheumatoid Arthritis	2021	66	20	22	10
15	Self-protection strategies and health behaviour in patients with inflammatory rheumatic diseases during the COVID-19 pandemic: results and predictors in more than 12 000 patients with inflammatory rheumatic diseases followed in the Danish DANBIO registry	2021	41	20	13.66667	9
16	Different COVID-19 outcomes among systemic rheumatic diseases: a nation-wide cohort study	2023	14	36	14	9
17	DMARD disruption, rheumatic disease flare, and prolonged COVID-19 symptom duration after acute COVID-19 among patients with rheumatic disease: A prospective study	2022	26	42	13	8
18	COVID-19 vaccination in autoimmune diseases (COVAD) study: vaccine safety and tolerance in rheumatoid arthritis	2023	13	22	13	8
19	Local and systemic reactogenicity of COVID-19 vaccine BNT162b2 in patients with systemic lupus erythematosus and rheumatoid arthritis	2021	35	12	11.66667	7
20	Non-invasive vagus nerve stimulation for COVID-19: results from a randomized controlled trial (SAVIOR I)	2022	23	49	11.5	7
21	Autoimmune inflammatory rheumatic diseases post-COVID-19 vaccination	2022	18	37	9	6
22	Four SARS-CoV-2 vaccine doses or hybrid immunity in patients on immunosuppressive therapies: a Norwegian cohort study	2023	10	39	10	6
23	Is vaccination against COVID-19 associated with autoimmune rheumatic disease flare? A self-controlled case series analysis	2023	9	17	9	6
24	Withholding methotrexate after vaccination with ChAdOx1 nCov19 in patients with rheumatoid or psoriatic arthritis in India (MIVAC I and II): results of two, parallel, assessor-masked, randomised controlled trials	2022	17	31	8.5	5
25	SARS-CoV-2 outbreak in immune-mediated inflammatory diseases: the Euro-COVIMID multicentre cross-sectional study	2021	24	29	8	5
26	COVID-19 infection and hospitalization risk according to vaccination status and DMARD treatment in patients with rheumatoid arthritis	2022	16	38	8	5
27	Distinct impact of DMARD combination and monotherapy in immunogenicity of an inactivated SARS-CoV-2 vaccine in rheumatoid arthritis	2022	15	48	7.5	5
28	Prolonged COVID-19 symptom duration in people with systemic autoimmune rheumatic diseases: results from the COVID-19 Global Rheumatology Alliance Vaccine Survey	2022	15	37	7.5	5
29	Rheumatoid arthritis, psoriatic arthritis, and axial spondyloarthritis epidemiology in England from 2004 to 2020: An observational study using primary care electronic health record data	2022	11	39	5.5	4
30	Varicella zoster virus reactivation following COVID-19 vaccination in patients with autoimmune inflammatory rheumatic diseases: A cross-sectional Chinese study of 318 cases	2023	7	35	7	4
31	COVID-19 Vaccination Uptake Among Individuals With Immune-mediated Inflammatory Diseases in Ontario, Canada, Between December 2020 and October 2021: A Population-based Analysis	2022	11	23	5.5	4
32	Humoral response to coronavirus disease-19 vaccines is dependent on dosage and timing of rituximab in patients with rheumatoid arthritis	2022	11	16	5.5	4
33	Effectiveness of SARS-CoV-2 vaccination in patients with rheumatoid arthritis (RA) on DMARDs: as determined by antibody and T cell responses	2022	14	13	7	4
34	Acceptability of Vaccines Against Preventable Infections Including Coronavirus Disease 2019 Among Patients With Rheumatic Disease	2022	13	17	6.5	4
35	Strong response after fourth dose of mRNA COVID-19 vaccine in autoimmune rheumatic diseases patients with poor response to inactivated vaccine	2022	13	17	6.5	4
