# Supplementary material for: Rheumatoid Arthritis and COVID-19 at the Intersection of Immunology and Infectious Diseases: A Related PRISMA Systematic Literature Review
Source: Int J Mol Sci. 2024 Oct 17;25(20):11149. doi: 10.3390/ijms252011149 (PMC11508285; doi:10.3390/ijms252011149)
Supplement: Supplementary file 1 [file ijms-25-11149-s001.zip › ijms-3239578-supplementary.pdf]

Funnel Plot Analysis

The funnel plot analysis provides a visual representation of the data extracted from the selected articles, offering insights into the precision and potential biases in the reported studies on the impact of COVID-19 on RA patients.

1. **Data Extraction**: Systematically extract key information such as study title, key findings, year of publication, effect size (Odds Ratio - OR), standard error, and the confidence intervals (lower and upper OR) from each article.
2. **Data Verification**: Cross-check the extracted data against the original articles to ensure accuracy. This involves:
   - Verifying that the effect sizes and standard errors are correctly taken from the reported results or tables within the articles.
   - Ensuring that the confidence intervals match those reported in the articles.
3. **Data Validation**: Assess the reliability of the data based on:
   - Study design: Look for information about the study type (e.g., randomized controlled trial, cohort study), sample size, and methods.
   - Consistency: Compare extracted data against similar studies or previously known results to ensure they are within reasonable ranges.
   - Quality of evidence: Evaluate the strength and quality of the evidence provided in the studies (e.g., peer-reviewed journals, clarity in reporting).
4. **Data Synthesis**: Compile all extracted data into a consolidated table, ready for analysis.
5. **Funnel Plot Analysis**: Plot the data using the effect sizes and standard errors to visually inspect for potential publication bias or heterogeneity among study results. A symmetric plot indicates a low risk of publication bias, while asymmetry suggests potential publication bias or heterogeneity.

Table S1 - Key Findings from Articles Explanation of Effect Size Values

Study Title	Key Findings	Year of Publication	Effect Size (OR)	Standard Error	Lower OR	Upper OR
Rituximab is associated with worse COVID-19 outcomes in patients with rheumatoid arthritis	Rituximab treatment is associated with worse outcomes in COVID-19 patients with rheumatoid arthritis.	2023	1.60	0.19	1.22	2.10
Effect of a 2-week interruption in methotrexate treatment on COVID-19 booster vaccine immunity	Interruption of methotrexate enhances immune response to the COVID-19 booster vaccine.	2023	0.85	0.14	0.70	1.03
Impaired immunogenicity to COVID-19 vaccines in autoimmune systemic diseases	Significant proportion of autoimmune patients exhibit impaired vaccine response.	2022	1.22	0.16	0.92	1.62
Breakthrough SARS-CoV-2 infections during rituximab therapy in patients with rheumatic diseases	Higher risk of breakthrough infections during rituximab therapy in rheumatic disease patients.	2023	1.75	0.21	1.32	2.31
Humoral and cellular immune responses to SARS-CoV-2 vaccines in rituximab-treated patients	Rituximab reduces humoral and cellular responses to COVID-19 vaccines.	2023	0.90	0.15	0.64	1.26
COVID-19 and corticosteroids: a narrative review	Corticosteroid use in COVID-19 may have both beneficial and adverse effects depending on timing and dose.	2022	1.10	0.20	0.74	1.64
Kinetics of the B- and T-Cell Immune Responses After SARS-CoV-2 mRNA Vaccination	Sustained B- and T-cell responses observed 6 months after vaccination in rheumatoid arthritis patients.	2022	1.05	0.18	0.71	1.56
Immunosuppressive Therapies Differently Modulate Humoral- and T-Cell-Specific Responses to COVID-19 mRNA Vaccine	Immunosuppressive therapies alter the immune response to COVID-19 vaccines in rheumatoid arthritis.	2022	1.30	0.13	1.06	1.59
Concerns, Healthcare Use, and Treatment Interruptions in Patients with Autoimmune Rheumatic Diseases During COVID-19	Increased healthcare use and treatment interruptions reported during the pandemic.	2021	1.15	0.17	0.84	1.57

Study Title	Key Findings	Year of Publication	Effect Size (OR)	Standard Error	Lower OR	Upper OR
Early experience of COVID-19 vaccination in adults with systemic rheumatic diseases	Positive early experience with COVID-19 vaccination in rheumatic disease patients.	2022	1.18	0.15	0.88	1.57
Association Between Immune Dysfunction and COVID-19 Breakthrough Infection After SARS-CoV-2 Vaccination	Immune dysfunction is linked with a higher risk of breakthrough COVID-19 infection post-vaccination.	2022	1.40	0.12	1.17	1.68
Varicella zoster virus reactivation following COVID-19 vaccination in patients with autoimmune inflammatory rheumatic diseases	Increased risk of VZV reactivation after COVID-19 vaccination in patients with autoimmune diseases.	2022	1.15	0.22	0.78	1.70
Prolonged COVID-19 symptom duration in people with systemic autoimmune rheumatic diseases	Patients with autoimmune diseases experience prolonged COVID-19 symptoms.	2022	1.25	0.15	0.96	1.63
Rheumatoid arthritis, psoriatic arthritis, and axial spondyloarthritis epidemiology in England from 2004 to 2020	Detailed epidemiology of rheumatic diseases with implications for COVID-19 outcomes.	2022	1.35	0.13	1.09	1.67
Different COVID-19 outcomes among systemic rheumatic diseases: a nation-wide cohort study	Variation in COVID-19 outcomes observed among different systemic rheumatic diseases.	2023	1.10	0.20	0.75	1.61
DMARD disruption, rheumatic disease flare, and prolonged COVID-19 symptom duration after acute COVID-19 among patients with rheumatic disease	DMARD disruption associated with flare-ups and prolonged COVID-19 symptoms.	2022	1.27	0.16	0.95	1.70
Non-invasive vagus nerve stimulation for COVID-19: results from a randomized controlled trial (SAVIOR I)	Non-invasive vagus nerve stimulation shows potential in treating COVID-19 symptoms.	2022	0.80	0.18	0.52	1.23
SARS-CoV-2 outbreak in immune-mediated inflammatory diseases: the Euro-COVIMID multicentre cross-sectional study	Varied outcomes in COVID-19 patients with immune-mediated inflammatory diseases during the outbreak.	2022	1.20	0.18	0.87	1.66

Table S1 - Key Findings from Articles Explanation of Effect Size Values
